# Catastrophic stroke burden in a patient with uncontrolled psoriasis and psoriatic arthritis: a case report

**DOI:** 10.1186/s12883-020-01681-9

**Published:** 2020-03-21

**Authors:** Joline M. Fan, David A. Solomon, Giselle Y. López, Jeffrey W. Hofmann, Rene A. Colorado, Anthony S. Kim, Karl Meisel, Cathra Halabi

**Affiliations:** 1 Department of Neurology, University of California, San Francisco, California USA; 2 Division of Neuropathology, Department of Pathology, University of California, San Francisco, California USA; 3 Division of Neuropathology, Department of Pathology, Duke University, Durham, North Carolina USA; 4 Department of Neurology, Salinas Valley Memorial Healthcare System, Salinas, California USA; 5 Weill Institute for Neurosciences, University of California, San Francisco, California USA

**Keywords:** Atherosclerosis, Stroke, Psoriasis, Psoriatic arthritis

## Abstract

**Background:**

Psoriasis is the most common chronic inflammatory condition involving the T helper cell system. Population studies have demonstrated that patients with psoriasis and/or psoriatic arthritis have an increased risk of developing vascular risk factors, including diabetes, hypertension, and obesity, and increased risk of adverse vascular events, including myocardial infarction and stroke. Population studies have generally investigated the individual contributions of psoriasis and psoriatic arthritis to development of vascular risk factors; fewer studies have investigated the additive contribution of comorbid inflammatory disorders. We present a case of a woman with psoriasis, psoriatic arthritis, and comorbid vascular risk factors.

**Case presentation:**

A 49 year-old Caucasian woman with a history of severe psoriasis and psoriatic arthritis since adolescence presented with bilateral lower extremity weakness. She was found to have acute bilateral watershed infarcts and multifocal subacute infarcts. Her evaluation revealed vascular risk factors and elevated non-specific systemic inflammatory markers; serum and cerebral spinal fluid did not reveal underlying infection, hypercoagulable state, or vasculitis. Over the course of days, she exhibited precipitous clinical deterioration related to multiple large vessel occlusions, including the bilateral anterior cerebral arteries and the left middle cerebral artery. Autopsy revealed acute thrombi and diffuse, severe atherosclerosis.

**Conclusion:**

Patients with early onset inflammatory disease activity or comorbid inflammatory disorders may have an even higher risk of developing metabolic syndrome and adverse vascular events compared to patients with late-onset disease activity or with a single inflammatory condition. The described case illustrates the complex relationship between inflammatory disorders and vascular risk factors. The degree of systemic inflammation, as measured by severity of disease activity, has been shown to have a dose-response relationship with comorbid vascular risk factors and vascular events. Dysregulation of the Th1 and Th17 system has been implicated in the development of atherosclerosis and may explain the severe atherosclerosis seen in such chronic inflammatory conditions. Further research will help refine screening and management guidelines to account for comorbid inflammatory disorders and related disease severity.

## Background

Psoriasis is a common chronic inflammatory disorder affecting approximately 1.5–3% of the adult population [1, 2]. An additional 6–30% of patients with psoriasis also have psoriatic arthritis, which may reflect a more pronounced systemic disease [3–5]. Population cohort studies have identified both psoriasis and psoriatic arthritis to be individual risk factors for vascular disease [4–7]; however, the contribution of comorbid inflammatory diseases for clinical screening and management guidelines remains unknown. Here, we present an illustrative report of a fatal stroke in a young patient with severe psoriasis, psoriatic arthritis, and metabolic syndrome.

## Case presentation

A 49 year-old Caucasian woman with psoriasis, psoriatic arthritis, multivessel coronary artery disease, hypertension, subclinical hypothyroidism, and diabetes mellitus presented with bilateral lower extremity weakness and severe anemia. Regarding her history of psoriasis, she initially developed diffuse psoriatic plaques and axial psoriatic arthritis at age 19. Her first severe psoriasis flare occurred at age 29. Chart review did not reveal recorded Psoriasis Area and Severity Index (PASI) scores but there was documentation of erythema and pustular psoriasis measured over 70% of her body surface area with elevated white blood cell count. Despite treatment with prednisone and acitretin, after 1 year she developed severe cutaneous flares of pustular psoriasis measuring up to 90% of total body surface area with spared regions in her legs, necessitating multiple hospitalizations. Her treatment was escalated to Geockerman therapy, methotrexate, and topical steroids, in addition to prednisone and acitretin. In subsequent years, her chronic psoriatic skin manifestations involved roughly 30% of her total body surface area, meeting criteria for severe psoriasis, and these skin manifestations were managed primarily with topical steroids. Plain films ultimately revealed active inflammatory spondylitis. Despite recommendations to start disease modifying therapy, the patient declined further treatment.

Six years prior to the most recent presentation, the patient was diagnosed with metabolic syndrome. Risk factors measured at the time of diagnosis included a peak hemoglobin A1c level of 11.6%, body mass index of 36, triglycerides of 310, high density lipoprotein (HDL) of 34, and systolic blood pressures routinely measured between 140 and 160. For her modifiable risk factors of diabetes, dyslipidemia, and hypertension, she was prescribed insulin, statins, and antihypertensive agents, respectively. Her family history was notable for ischemic stroke in her mother though of unknown etiology and age. There were no known inflammatory disorders in the family. Three months prior to the most recent presentation, the patient was hospitalized for a non-ST elevation myocardial infarction (NSTEMI). Cardiac catheterization revealed severe right coronary artery (RCA) disease and in-stent thrombosis of a pre-existing stent within the left circumflex artery placed 6 years prior. She underwent re-stenting for her left circumflex artery and RCA and was subsequently treated with a dual antiplatelet therapy regimen with aspirin and clopidogrel.

**Fig. 1:**
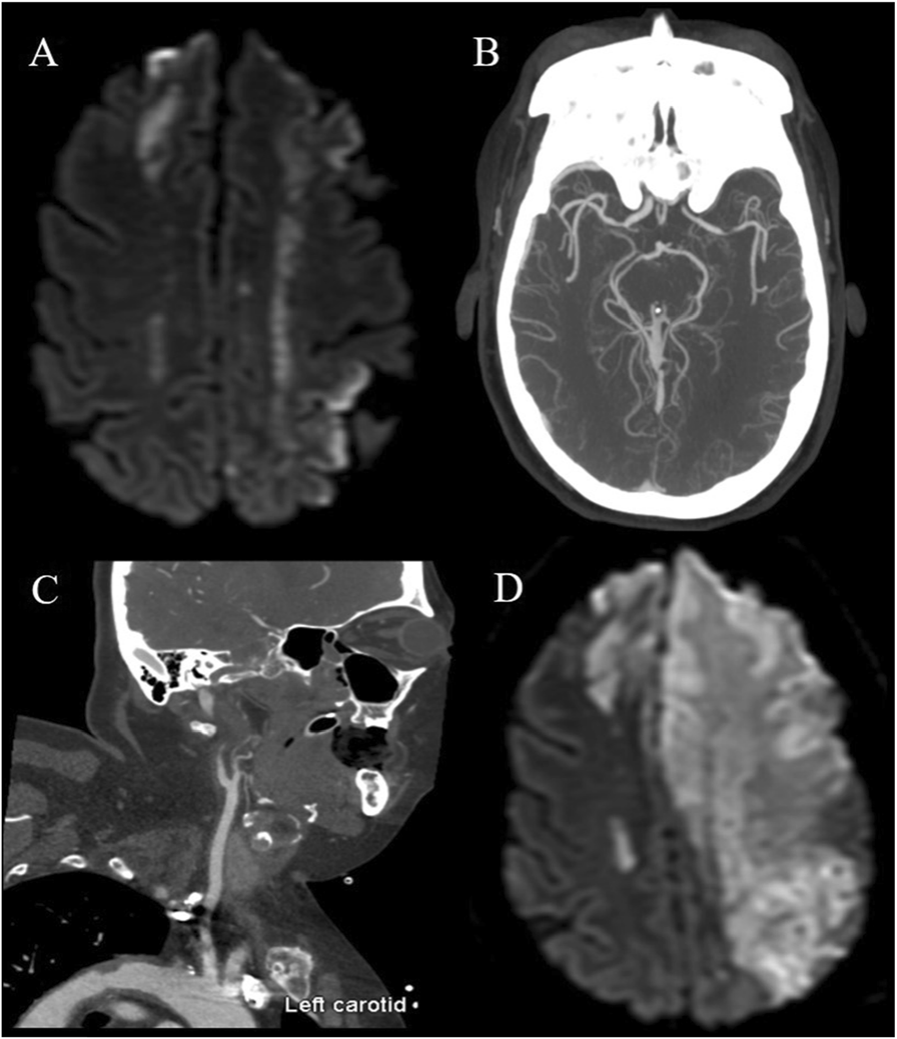
**a** Diffusion-weighted axial MRI showing bilateral anterior cerebral artery (ACA) and middle cerebral artery (MCA) distribution infarcts. **b** CTA axial MIP demonstrating mild irregular luminal narrowing of the right M1 segment and severe focal narrowing of the distal left M1 segment. c CTA sagittal MIP demonstrating calcified atherosclerotic plaque of the left carotid bulb and complete occlusion of the proximal cervical left internal carotid artery. **d** Diffusion-weighted axial MRI showing marked interval progression of infarcts involving the bilateral ACA and left MCA distributions

Subsequently, she presented with subacute bilateral lower extremity weakness and confusion. Her brain MRI revealed acute bilateral watershed infarcts, in addition to subacute left parietal and frontal gyrus infarcts. CT angiography of the head and neck revealed diffuse atherosclerotic plaques in the aortic arch and carotid bulbs, occlusion of the left internal carotid artery (ICA), and narrowing of the bilateral M1 segments of the middle cerebral artery (MCA). (Fig. 1a-c). There was reconstitution of flow in the left cavernous and supraclinoid segments of the ICA via an intact Circle of Willis. In addition to the bilateral MCA narrowing, there was mild narrowing of the right carotid artery and a single diminutive A1 segment which perfused bilateral anterior cerebral arteries (ACA) vessels.

There were no known preceding hemodynamic changes or systemic illnesses. Possible etiologies of her multifocal infarcts included severe atherosclerosis and related artery-artery thromboembolic events, cardioembolic events, hypercoagulable state, infection, or vasculitis. Cardiac rhythm telemetry did not reveal atrial fibrillation. Transthoracic echocardiogram revealed normal left ventricular function, normal left and right atrial size, no thrombus, and no intra- or extra-cardiac shunt. Subsequent transesophageal echocardiogram (TEE) again did not reveal thrombus or valvular lesions. The TEE also demonstrated atheromatous plaque in the proximal descending aorta greater than 4 mm in thickness. Laboratory studies included hemoglobin (Hg) of 4.2 g/dL, an elevated erythrocyte sedimentation rate > 100 mm/hr. (normal 0–15 mm/hr) and lipoprotein (a) 133 nmol/L (normal < 75 nmol/L) but were otherwise unrevealing (Table 1). The cerebral spinal fluid (CSF) did not have infectious or inflammatory properties. Workup of anemia was consistent with alpha thalassemia and severe iron deficiency, also supported by peripheral blood smear.

**Table 1:** Summary of serum and CSF studies obtained on present hospitalization

	Data	Reference
**Lipid profile**		
Cholesterol, Total	179	< 200 mg/dL
Triglycerides	253	< 200 mg/dL
LDL	114	< 130 mg/dL
Lipoprotein a	133	< 75 nmol/L
**Hypercoagulation**		
Beta-2-glycoprotein Antibody, IgG	< 10.0	< 20.1 CU
Beta-2-glycoprotein Antibody, IgM	< 5.0	< 20.1 CU
Protein C, Activity	99	76–146%
Anti-Cardiolipin Antibody, IgG	15.8	< 20.1 CU
Anti-Cardiolipin Antibody, IgM	< 10.0	< 20.1 CU
Lupus Anticoagulant by HEXA	Neg	Neg
RVVT Seconds	30.9	29.0–44.0 s
**CSF studies**		
WBC	1	< 6 x10E6/L
RBC	44	0 x10E6/L
Glucose	76	40–70 mg/dL
Protein	13	15–50 mg/dL
IgG Index	0.6	0.3–0.6 Ratio
Serum glucose	164	< 200
Rapid HSV-1 PCR	Neg	Neg
Rapid HSV-2 PCR	Neg	Neg
VZV PCR	Neg	Neg
VZV IgM	0.03	< 0.9
**Autoimmune/Endocrine**		
Hemoglobin A1c	6.4	4.3–5.6%
TSH	0.64	0.45–4.12 mlU/L
Free T4	13	10–18 pmol/L
CRP	5.4 - > 36.9	< 6.3
ESR	> 100	0–15 mm/h
Anti-Nuclear Antibodies	< 40	< 40
Anti Proteinase 3 Ab	< 10.0	< 20.0 CU
Anti Myeloperoxidase	< 10.0	< 20.0 CU
GAD-65 Autoantibodies	< 1.0	< 1.0 U/mL
Insulin Autoantibody	< 0.4	< 0.4 U/mL
Islet Cell Antigen 512 Antibody	< 0.8	< 0.8 U/mL
Rheumatoid Factor, serum	< 40	< 40 IU/mL
Complement C3, serum	101	71–159 mg/dL
Complement C4, serum	26.4	13–30 mg/dL
Cryoglobulin	< 0.12	< 0.12 g/L
Heterophile Agglutination	Neg	Neg
**Infectious**		
Hepatitis B sAb	< 5	mIU/mL
Hepatitis B cAb	Neg	Neg
Hepatitis B sAg	Neg	Neg
Hepatitis C Antibody	Neg	Neg
HIV Ag/Ab	Neg	Neg

Despite aggressive medical management, the patient clinically deteriorated. Repeat imaging revealed marked interval infarct progression involving multiple vascular territories (Fig. 1d). She underwent a left-sided decompressive hemicraniectomy as a life sustaining maneuver, and concomitant brain biopsy did not demonstrate evidence of vasculitis or infection. Clinical course was further complicated by sepsis, renal failure, and an acute generalized exanthematous pustulosis drug reaction to iodine contrast. In accordance with the patient’s prior wishes, she was transitioned to comfort-focused care and her family agreed to an autopsy.

**Fig. 2:**
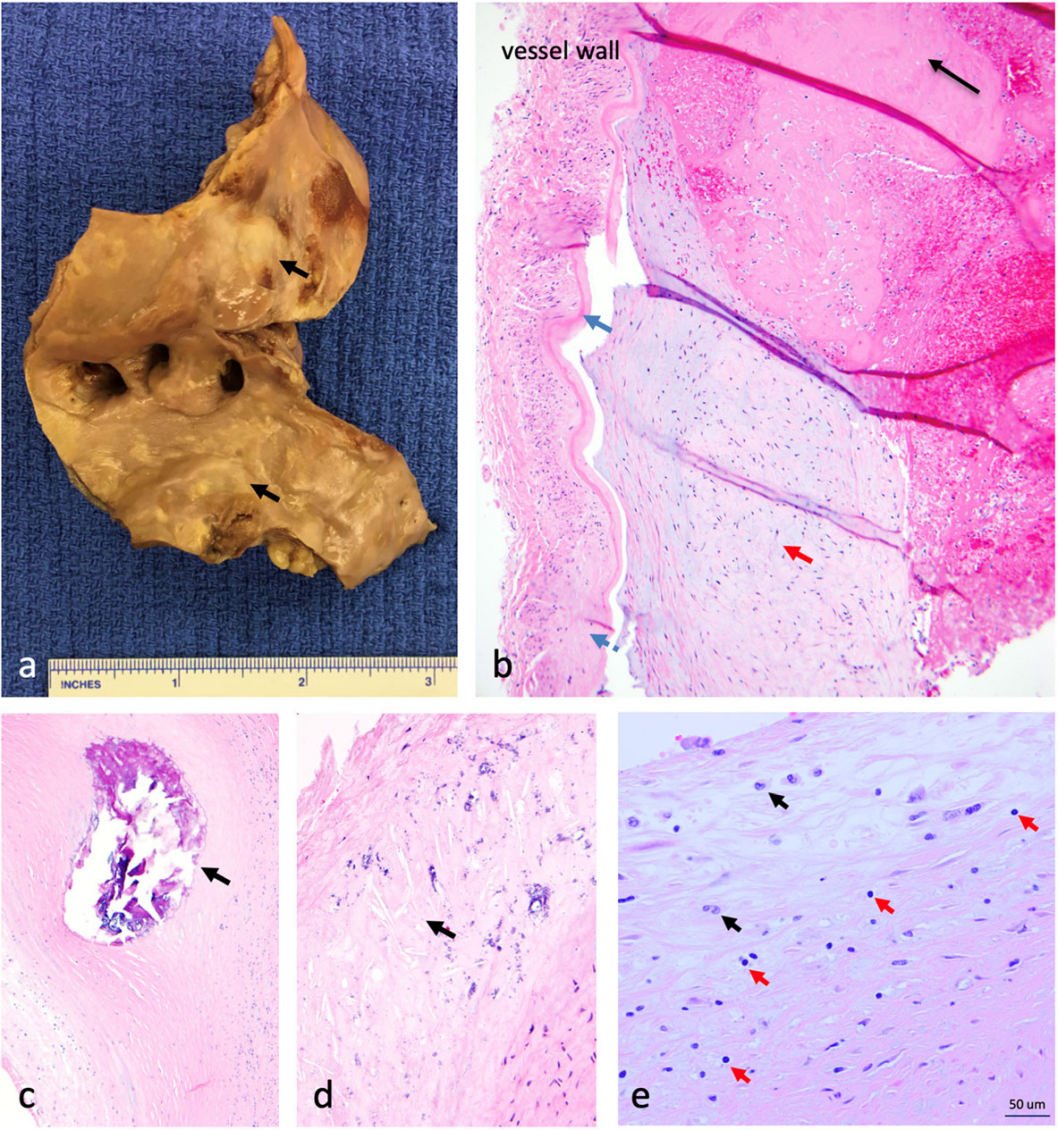
Gross and microscopic pathology. **a** Gross pathology of aortic arch revealing extensive atheromas (black arrows). **b** Histopathology of the anterior cerebral artery. There is intimal thickening underlying the internal elastic lamina (blue arrow); the media and adventitia are distorted by fibrosis (blue dashed arrow). A recently-formed fibrin thrombus (black arrow) is adherent to a chronic and organized atherosclerotic plaque (red arrow). **c** Cross-sectional view of the left anterior descending coronary artery containing an intramural calcified nodule (black arrow). **d** Cross-sectional view of the basilar artery wall, demonstrating architectural distortion with cholesterol clefts (black arrow) and microcalcifications. **e** Left anterior descending artery with thickening of the intima and inflammatory infiltrate including T lymphocytes (red arrows) and macrophages (black arrows)

The autopsy revealed eccentric, firm, yellow plaques involving all proximal intracranial vessels, consistent with severe atherosclerosis. There was an occlusive thrombus of the right ACA as well as diffuse arteriolar microthrombi. Elements of acute and chronic ischemic changes were seen on histology, including extensive ischemic neuronal necrosis. Diffuse, severe atherosclerosis was determined to be the cause of fatal burden of ischemic stroke (Fig. 2).

## Discussion and conclusions

We present a case of a young patient with psoriasis and psoriatic arthritis, severe and diffuse atherosclerosis, and ultimately a fatal burden of ischemic infarcts of the brain. While the definitive contributions of the patient’s individual inflammatory conditions and anemia are unknown, we suggest that severe psoriasis and psoriatic arthritis enhance and accelerate progression of early presence of vascular risk factors and that recognition of this relationship provides another opportunity for risk factor stratification and treatment. Psoriasis and psoriatic arthritis have each been associated with cardiovascular risk factors and metabolic syndrome. This report illustrates that the burden and severity of comorbid inflammatory conditions and metabolic syndrome should prompt aggressive screening and management of vascular risk factors.

### Pathophysiology

The pathophysiology of psoriasis involves the activation of effector T lymphocytes, including T helper cell type 1 and 17. Skin infiltration of activated T cells promotes antigen presentation and propagation of inflammatory cytokines [3]. A dysregulated Th1 system within the vessel wall intimal layer is implicated in the development of atherosclerosis and is a biologically plausible explanation for accelerated atherosclerosis in a chronic inflammatory state [8, 9]. The initial lesion responsible for atherogenesis is the fatty-streak, which is pathologically composed of monocytes and T lymphocytes [8, 10]. Endothelial cell activation leads to an enhanced local inflammatory response with upregulation of leukocyte adhesion molecules, increased of leukocyte recruitment, and expansion of the subendothelial fatty streak, leading to plaque formation [10, 11]. Progressive focal narrowing of vessels is punctuated by exposure of thrombogenic material when there is intimal layer plaque rupture or endothelial cell layer disruption. Thrombosis then leads to conditions such as unstable angina, myocardial infarction, or stroke [11, 12]. Fig. 2 illustrates extensive atheromas (2a) and chronic changes to the vessel wall layers (2c-d). Figure 2e illustrates the presence of T lymphocytes and macrophages within the thickened intima, which can be seen within the natural process of atherosclerosis [8, 10]. There is no evidence of excessive or patterned presence of inflammatory cells in pathology specimens, suggesting a chronic, cumulative process, and highlighting indirect and potentially synergistic roles of systemic inflammation in accelerating atherosclerosis.

Circulation of inflammatory substances including low density lipoprotein (LDL) and lipoprotein (a) have also been directly implicated in vessel wall reactivity and plaque formation [3]. Studies have shown increased subclinical atherosclerosis and increased intima-medial thickness in patients with psoriatic arthritis, but without otherwise clear cardiovascular risk factors [13, 14], illustrating a more direct effect of systemic inflammation on the arterial wall. More severe subclinical atherosclerosis has also been seen in patients with psoriatic arthritis as compared to control populations and patients with cutaneous psoriasis alone [12]. The severity of atherosclerosis may increase with the added burden of inflammatory joint disease in patients with psoriasis.

### Psoriasis

Population studies have demonstrated that patients with psoriasis have increased cardiovascular risk factors, including diabetes, hypertension, hyperlipidemia, and obesity, which are all features of metabolic syndrome [15–18]. Across observational studies, meta-analyses describe a 1.4–2.2 fold increased risk of developing metabolic syndrome in patients with psoriasis as compared to controls [18, 19]. In addition, the risk of developing metabolic syndrome has been shown to increase with psoriasis severity, with odds ratios (OR) of 1.22, 1.56, 1.98 for mild, moderate, and severe psoriasis, respectively [18]. Other studies have noted specific risk factors that are more prevalent in severe versus mild psoriasis, including diabetes (OR 1.39, 95% CI 1.22–1.58) and obesity (OR 1.47, 95% CI 1.32–1.63) [20]. These studies illustrate the relationship between the degree of systemic inflammation and development of metabolic syndrome.

In addition to the added risk conferred from elevated cardiovascular risk factors, population studies have also demonstrated that psoriasis independently leads to an increased risk of myocardial infarction or stroke [7, 18, 21, 22]. After adjusting for cardiovascular risk factors, studies demonstrate that mild and severe psoriasis confer a 1.29-fold risk (95% CI 1.02–1.63) and a 1.7-fold risk (CI 1.32–2.18) of myocardial infarction, as well as a 1.12-fold risk (95% CI, 1.08–1.16) and a 1.56-fold risk (95% CI 1.32–1.84) of stroke [23], respectively. These findings suggest a shared inflammatory pathway involved in the pathogenesis of psoriasis and atherogenesis [24].

The risk of cardiovascular risk factors and adverse events has also been noted to correlate with age of onset [22, 25]. The risk of incident myocardial infarction (MI) was found to be higher in younger patients with severe psoriasis as compared to older patients with late-onset psoriasis (HR 3.10 in a 30 year-old vs 1.36 in a 60 year-old) [25]. The difference in the disease activity of psoriasis is dependent on both genetic and environmental factors. Detection of specific human leukocyte antigens (HLA), such as type Cw6, has been differentially found in patients with early onset psoriasis as compared to late onset psoriasis, as one example of biological differences that contribute towards disease activity [26].

### Psoriatic arthritis

Other studies have looked at the contribution of psoriatic arthritis as an independent risk factor for metabolic syndrome and adverse cardiovascular events. Patients with psoriatic arthritis have increased prevalence of hypertension (OR 1.31, 95% CI 1.26–1.47), hyperlipidemia (OR 1.23, 95% CI 1.18–1.29), diabetes (OR, 1.38, 95% CI 1.31–1.45), and obesity (OR 1.69, 95% CI 1.62–1.75) [4]. The prevalence of MI (OR 1.36-fold, 95% 1.04–1.77) and stroke (OR 1.26-fold, 95% CI 1.03–1.71) was also higher in patients with psoriatic arthritis as compared to controls [7, 27]. Notably, diagnosis of psoriatic arthritis did not confer a statistically significant elevation in mortality, as compared to controls [5]. Fewer studies have looked at the additive risk of accruing vascular risk factors in patients that have psoriatic arthritis in addition to cutaneous psoriasis. One study reports the elevated risk of vascular risk factors for patients with psoriatic arthritis to be 2.59-fold (95% CI 1.43–4.67) for cardiovascular disease, 2.42-fold (95% CI, 1.82–3.22) for hypertension, 1.9-fold (95% CI, 1.22–2.96) for diabetes, and 1.58-fold (95% CI, 1.19–2.09) for obesity [28, 29]. The presence of multiple inflammatory conditions may imply presence of multiple vascular risk factors.

There are many contributing factors toward atherosclerosis and vascular events, including conventional risk factors of dyslipidemia and hypertension, family history, ethnicity, and lifestyle [30]. The potential impact of anemia [31] and hemoglobinopathy [32] is also acknowledged but not definitive. In this report, however, we have focused on addressing the direct and indirect contributions of psoriasis and psoriatic arthritis.

### Immunosuppressive therapy

Methotrexate and tumor necrosis factor inhibitors lead to 0.72-fold (95% CI 0.57–0.91) and 0.7-fold (95% CI 0.54–0.9) respective reduction of incident vascular events including MI and stroke in rheumatic diseases while agents such as cyclosporine, oral retinoids, steroids, and non-steroidal anti-inflammatory agents may confer increased risk [33, 34]. While fewer studies have been performed in patients with psoriasis and psoriatic arthritis, data suggests that disease modifying therapies, biologics, and anti-inflammatory agents may also lead to a reduced rate of cerebrovascular risk factors and events [35–39].

In conclusion, psoriasis and psoriatic arthritis have both indirect and direct causative roles in increasing the risk of vascular disease. Younger patients or those with multisystem disease need aggressive screening and treatment for active inflammatory disease along with vascular risk factor stratification. In patients with inflammatory conditions, we suggest screening for modifiable vascular risk factors and treating the risk factors in accordance with contemporary guidelines. Similarly, in young patients with vascular risk factors, we suggest reviewing systems for undiagnosed inflammatory disease in the appropriate clinical context. Studies are needed to help identify appropriate immunosuppressive therapy selection for synergistic risk factor reduction. Studies are also needed to help identify timing, cost-effectiveness, and patient selection for screening and treatment strategies.

## Data Availability

All data generated or analyzed during this study are included in this published article.

## Abbreviations

ACA: Anterior cerebral artery
CI: Confidence interval
CTA: Computerized tomography angiogram
HDL: High-density lipoprotein
Hg: Hemoglobin
HLA: Human leukocyte antigens
HR: Hazard ratio
ICA: Internal carotid artery
LDL: Low density lipoprotein
MCA: Middle cerebral artery
MI: Myocardial infarction
NSTEMI: Non-ST elevation myocardial infarction
OR: Odds ratios
PASI: Psoriasis Area and Severity Index
RCA: Right coronary artery
TEE: Transesophageal echocardiogram
